# Agreement between fingertip-capillary and antecubital-venous appetite-related peptides

**DOI:** 10.1530/EC-14-0110

**Published:** 2014-10-28

**Authors:** Benjamin Paul Green, Javier Thomas Gonzalez, Kevin Thomas, Emma Stevenson, Penny Louise Sheena Rumbold

**Affiliations:** 1 Department of Sport, Exercise and Rehabilitation, Faculty of Health and Life Sciences, Northumbria University, Northumberland Building, Newcastle upon Tyne NE1 8ST, UK

**Keywords:** GLP1_7–36_, glucagon, insulin and leptin

## Abstract

This study examined the agreement between fingertip-capillary and antecubital-venous measures of appetite-related peptides. Simultaneous fingertip-capillary and antecubital-venous blood samples were collected from 19 participants. The samples were obtained at baseline, 30, 60, 90, and 120 min following breakfast for the determination of plasma GLP1_7–36_, glucagon, insulin and leptin. Between-day reproducibility of fingertip-capillary-derived estimates was assessed in 18 participants. Deming regression, limits of agreement (LOA) and typical error as a coefficient of variation (CV) were used to quantify agreement (CV_a_) and reproducibility (CV_r_). Deming regression revealed no systematic bias for any of the analytes studied, but for insulin there was evidence of a proportional difference at higher concentrations. Measures of GLP1_7–36_ (CV_a_=24.0%, LOA ±2.5 pg m/l per h), leptin (CV_a_=9.0%, LOA ×/÷1.19) and glucagon (CV_a_=21.0%, LOA, ±31.5 pg m/l per h) revealed good agreement between methodological approaches. Fingertip-capillary glucagon was highly reproducible between days (CV_r_=8.2%). GLP1_7–36_ and leptin demonstrated modest reproducibility (CV_r_=22.7 and 25.0% respectively). For insulin, agreement (CV_a_=36.0%, LOA ×/÷1.79) and reproducibility were poor (CV_r_=36.0%). Collectively, the data demonstrate that fingertip-capillary blood sampling provides a comparable and reproducible alternative to antecubital-venous blood sampling for the quantification of glucagon, and to a lesser extent for GLP1_7–36_ and leptin. Caution should be exercised when utilising fingertip-capillary blood sampling for insulin quantification, and consequently should not be employed interchangeably with antecubital-venous blood sampling.

## Introduction

Multiple hormonal peptides involved in the regulation of energy homeostasis are produced and secreted into the circulation in response to the ingestion of a meal. These peptides, emanating from the periphery of the gastrointestinal tract, pancreas, and adipose, act to influence the physiological mechanisms controlling energy regulation, eliciting divergent actions on feeding behaviour and metabolism (1). In humans, hormonal peptides such as glucagon-like peptide-1 (GLP1) (2), glucagon (3), insulin (4), and leptin (5), among others, represent several commonly measured metabolic variables documented as key effectors targeting energy intake and expenditure. Accurate quantification of these peptides is therefore essential when exploring hormonal responses in studies concerning appetite, feeding behaviour and metabolism.

In clinical and research practice, quantitative measures of appetite- and metabolism-related peptides have commonly been assessed using venepuncture or antecubital-venous catheterisation. Obtaining i.v. access is often problematic in certain exercise (particularly running) and field settings and with vulnerable populations, where alternative techniques for blood sampling may be effective. Fingertip-capillary blood sampling offers an alternative approach to venous sampling and may help to overcome issues associated with venepuncture or antecubital-venous catheterisation. Furthermore, the nature of fingertip-capillary blood sampling poses numerous advantages including simple application, reduced ethical consideration and volume of blood required for analysis (6). To date, only three studies have employed fingertip-capillary blood sampling for quantification of appetite- and metabolism-related peptides including leptin, insulin, and GLP1_7–36_ (7, 8, 9). Data from these studies indicate that fingertip-capillary-derived measures were representative of values reported from previous research employing antecubital-venous sampling, and begin to provide affirmation for the use of this approach in energy-related research.

No evidence exists, however, confirming fingertip-capillary-derived measures of appetite- and metabolism-related peptides are reproducible, and accurately reflect concentrations in comparison with their antecubital-venous equivalents. The test–retest reproducibility of these peptides is therefore essential, making certain that an intervention(s) or variable(s) is responsible for any observed differences and is not brought about by random variability or measurement error (10, 11). Quantification of agreement and reproducibility between methods would facilitate appropriate comparisons between studies using venous and capillary blood sampling. This is particularly important given that blood obtained from different sampling locations (e.g. antecubital-venous blood and fingertip-capillary blood) is characteristically dissimilar. Capillary blood, for example, encompasses an assortment of blood from venules, arterioles, intracellular and interstitial fluid and of course the capillaries. The quantity of arterialised blood is more pronounced in capillary blood compared with antecubital-venous blood, and is therefore thought to be more reflective of arterial blood (12).

Accordingly, the aims of this study were twofold. Firstly, the study was designed to examine the agreement of GLP1_7–36_, glucagon, insulin and leptin between fingertip-capillary blood sampling and antecubital-venous blood sampling in a resting state (part 1). Secondly, this study assessed the between-day test-retest reproducibility (part 2) of the aforementioned peptides in a resting state using fingertip-capillary blood sampling.

## Materials and methods

### Participants

In total, 19 healthy adult participants (15 males and four females) from the staff and student population of Northumbria University at Newcastle upon Tyne participated in part 1. Eighteen healthy adult participants (nine males and nine females) participated in part 2 of this study. Participant details are provided in Table 1. The participants were informed of the purpose, procedures and potential risks of the study and written informed consent was obtained from all before data collection. The study was conducted according to the guidelines laid down in the 2013 Declaration of Helsinki (13), and all procedures involving human subjects were approved by the Faculty of Health and Life Sciences Ethics Committee of the University of Northumbria.

**Table 1:** Participant characteristics by study.

	**Part 1**		**Part 2**	
	Agreement (*n*=19)		Between-day reproducibility (*n*=18)	
	Mean	s.d.	Mean	s.d.
Age (years)	24.1	5.7	23.1	3.5
Mass (kg)	73.7	10.9	69.3	12.7
Height (m)	1.8	0.1	1.7	0.1
BMI (kg/m^2^)	23.6	2.1	23.1	2.7

### Study design

In part 1, participants attended the laboratory on a single occasion where simultaneous samples of fingertip-capillary and antecubital-venous blood were collected to assess the agreement between the measures of appetite- and metabolism-related peptides. The samples were obtained in the fasting and postprandial state for the determination of plasma GLP1_7–36_, glucagon, insulin and leptin. For part 2, participants attended the laboratory on two separate occasions separated by 7 days, where fingertip-capillary blood was sampled to determine the reproducibility of the aforementioned peptides. The participants reported to the clinical testing laboratory at designated times between 0700 and 0900 h, following a 12 h overnight fast. The time participants attended the laboratory was documented and kept consistent between successive trials (part 2 only). The participants were instructed to refrain from the consumption of caffeine and alcohol (≥12 h) and strenuous physical activity (≥24 h) preceding data collection. Between waking and arrival at the clinical testing laboratory, consumption of water only was permitted. The participants were requested to record, document and replicate morning water consumption for subsequent trials. Following baseline (*t*=0) blood samples, participants were issued with a standardised cereal and milk breakfast. Further samples of antecubital-venous (part 1 only) and fingertip-capillary blood were collected at 30, 60, 90, and 120 min during the postprandial period. Additional food and beverage consumption was prohibited until test termination, apart from water that was offered *ad libitum*. In-trial *ad libitum* fluid consumption (if any) was documented and matched for subsequent trials. Throughout the test periods, participants remained sedentary in an environment free from food cues.

### Blood sampling

At five separate intervals, simultaneous fingertip-capillary (0.3 ml) and antecubital-venous blood samples (8.0 ml) were drawn into pre-cooled EDTA-treated microvettes and monovettes respectively. The samples were collected at baseline (*t*=0 min) and at 30, 60, 90, and 120 min following breakfast consumption for the determination of plasma GLP1_7–36_, glucagon, insulin and leptin. Venous blood was drawn from an indwelling cannula (Venflon 20G, Becton Dickinson & Company, East Rutherford, NJ, USA) inserted into an antecubital forearm vein. Patency of the cannula was preserved by flushing a small volume of non-heparinised saline (0.9% NaCl; Becton Dickinson & Company) through the connector tube on completion of each antecubital-venous sample. Residual saline waste was discarded immediately before succeeding sample points, avoiding contamination and dilution of antecubital-venous blood. Fingertip-capillary blood was simultaneously obtained from a pre-warmed fingertip pierced with a sterile automated lancet (Accu-Check, Mannheim, Germany). Fingertip-capillary blood samples were also collected for assessing reproducibility for part of the study. Approximately 3–5 min preceding each fingertip-capillary sample, the entire sample-hand was pre-warmed in warm water to promote an adequate flow of fingertip-capillary blood. On removal, the hand was dried thoroughly and the identified site for puncture was further cleansed by wiping with an aseptic alcohol. On puncturing the fingertip, the first drop of blood was removed before subsequent collection, where care was taken not to apply excessive pressure. All blood samples were obtained while subjects lay in a semi-supine position.

Blood collection tubes contained aprotinin (33 μl/ml blood) and a di-peptidyl peptidase IV inhibitor (30 μl/ml blood) for the preservation of GLP1_7–36_ and glucagon by proteases. Sample pre-treatment and the addition of protease inhibitors were applied to both microvettes and monovettes and performed preceding sample collection. Of note, the addition of protease inhibitors to plasma samples for the preservation of GLP1_7–36_ and glucagon does not influence measured concentrations of plasma leptin and insulin (14). Following collection, samples were placed on ice and immediately centrifuged. The monovettes were spun at 1509 ***g*** for 10 min at 4 °C in a refrigerated multispeed centrifuge and microvettes spun at 3000 r.p.m. for 10 min in a multispeed micro-centrifuge. The aliquots of plasma supernatant were housed in appropriately labelled eppendorfs and stored at −80 °C for the determination of plasma GLP1_7–36_, glucagon, insulin and leptin concentrations.

### Breakfast meal

Following baseline measures, participants consumed a standardised breakfast meal consisting of semi-skimmed milk (Sainsbury, London, UK) and Kellogg's Rice Krispies (Kelloggs, Manchester, UK), distributed at a cereal-to-milk ratio of 30 g: 125 ml. The quantity issued was designed to provide 10% of the participants estimated daily energy requirement for protein, fat and carbohydrate (14, 14, and 72% respectively) as previously used and drawn from recommendations from the National Diet and Nutrition Survey (15). Individual daily energy requirements were computed according to age and sex-specific calculations (16), providing an estimate of basal metabolic rate. The estimated values of basal metabolic rate were further multiplied against a self-perceived physical activity factor. The participants were given 15 min to consume the entire contents of the breakfast meal.

### Electrochemiluminescence

Quantitative assessments of GLP1_7–36_ (pg/ml), glucagon (pg/ml), leptin (pg/ml) and insulin (pmol/l) were simultaneously determined in duplicate in 40 μl of plasma by electrochemiluminescence using a human hormone multiplex assay kit (Sector Imager 2400, MesoScale Discovery, Rockville, MD, USA). To facilitate quantification, a standard curve was produced from a stock calibrator of known hormone concentration. The stock calibrator was provided by the manufacturer and diluted (fourfold serial dilutions) accordingly to generate an eight-point standard curve with a supplied ‘Metabolic Assay Working Solution’. As per manufacturer's instructions, the calibrators were analysed in duplicate and included for each set of unknown samples and on each assay plate. Using linear regression analysis, linearity and the quality of the curve-fit corresponded to *r* ^2^≥0.95 for all peptides and across all assays. The lower limit of detection (sensitivity) for GLP1_7–36_, glucagon, leptin and insulin was 0.7±0.2 pg/ml, 58.4±9.2 pg/ml, 101.6±6.8 pg/ml and 2.0±1.2 pmol/l respectively, as determined from an in-house analysis. The concentrations below the detection limit were left blank, yet accounted for within the time-averaged area under the curve (AUC) calculation. To eliminate inter-assay variation, samples from each participant were analysed within the same run. In part 1, average intra-assay coefficients of variation (CV) were 12, 7, 15, and 12% for GLP1_7–36_, glucagon, leptin and insulin respectively. For part 2, average intra-assay CV were 10, 8, 6, and 12% for GLP1_7–36_, glucagon, leptin and insulin respectively. Intra-assay CV were determined for part 1 by the repeated measurement of a single-baseline antecubital-venous plasma sample five times. For part 2, intra-assay CV were determined by the repeated measurement of a single-baseline fingertip-capillary plasma sample three times (primarily due to the reduced volume of plasma available for analysis).

### Statistical analyses

Descriptive data are presented as means±s.d. The time-averaged AUC score was computed for each peptide, in antecubital-venous and fingertip-capillary blood, using the trapezoidal rule. Agreement between fingertip-capillary and antecubital-venous-derived measurements was subsequently assessed using Deming regression (to test for systematic and proportional bias) (17), Bland–Altman (18) limits of agreement (LOA; to quantify random error), and typical error of the estimate as a CV (CV_a_) (%) to compare random error between measures (19). Assumptions of normal distribution and non-dependence of measurements were assessed using boxplots and scatter plots respectively (20). For Deming regression, systematic and proportional bias was evaluated by means of the intercept and slope respectively (21). For LOA, heteroscedasticity was assessed by inspecting scatter plots and associated Pearson's correlation coefficients of the absolute differences (errors) and measurement means (18). Where significant heteroscedasticity existed (defined as an *r *value >0.4), data were log transformed (natural) and reported as a geometric mean and ratio (×/÷) LOA. Statistical significance was accepted at *P*<0.05 for all analyses. Between-day test–retest reproducibility of fingertip-capillary measures was assessed using paired-samples *t*-tests and typical error as a CV (CV_r_) (%).

For the purpose of this study, pre-determined clinically significant differences were computed for each peptide before data collection. Differences employed were facilitated through literature-informed choices and their associated effects on subjective appetite, feeding behaviour and within-subject day-to-day biological variation. For plasma concentrations of GLP1_7–36_ and glucagon, pre-determined time-averaged AUC differences of 1.4 pg/ml per h (2) and 4.9 pg/ml per h (22), between methodological approaches and visits were deemed clinically important discrepancies respectively. Furthermore, differences of 62.0 pmol/l per h (23) and 148.0 pg/ml per h (24) were deemed clinically important for plasma concentrations of insulin and leptin between methodological approaches and visits respectively. Furthermore, based on the within-subject variation of antecubital-venous plasma glucagon (19% (25)), leptin (20% (24)) and insulin (26% (25)), typical error of the estimate when reported as a CV% was deemed strong when below 10%, modest when equal or similar to previously reported variation, and poor when substantially greater than this.

## Results

In total, 19 participants completed the agreement study. However, due to difficulties associated with cannulation and consequently blood collection, results for GLP1_7–36_, glucagon and insulin are presented for 17 subjects, and 16 subjects for leptin. All 18 participants successfully completed the between-day reproducibility study and the results for GLP1_7–36_, glucagon and insulin are provided. For leptin, however, results are available from 17 subjects. One participant's data were excluded on the basis of yielding a time-averaged AUC value of 3 s.d.s above the group mean.

### Agreement between antecubital-venous and fingertip-capillary-derived peptide hormones

**Figure 1:**
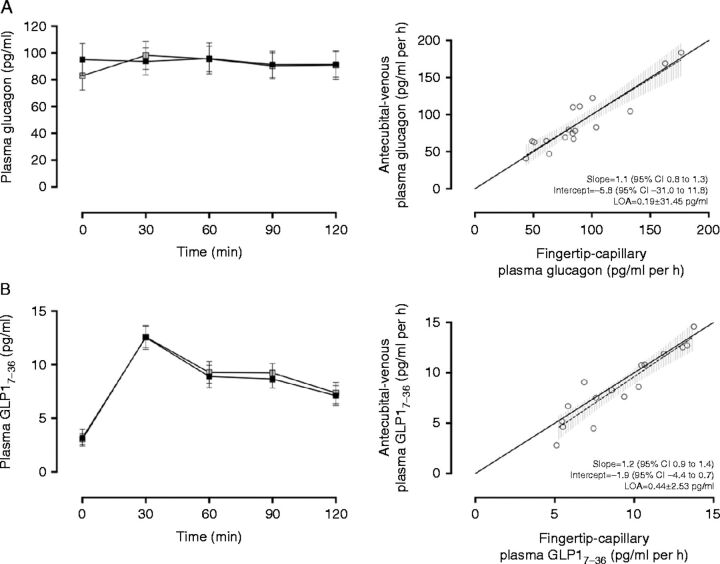
(A) Comparison of plasma glucagon (pg m/l) measures obtained simultaneously from fingertip-capillary and antecubital-venous blood samples (left). Deming regression scatter plot (right). The solid line represents the line of equality. The dashed line denotes the regression line with corresponding 95% CIs represented in the grey hashed area. (B) Comparison of plasma glucagon-like peptide- 1 (GLP1_7–36_) (pg m/l) obtained from simultaneous fingertip-capillary and antecubital-venous blood samples (left). Deming regression scatter plot (right). The solid line represents the line of equality. The dashed line denotes the regression line with corresponding 95% CIs represented in the grey hashed area. All values are expressed as mean±s.e.m. Grey shaded boxes represent values obtained from fingertip-capillary blood sampling, whereas black shaded boxes represent concentrations in antecubital-venous blood. To convert GLP1_7–36_ (pg m/l) and plasma glucagon (pg m/l) to their corresponding SI units multiply values by 0.298 and 0.287 respectively.

**Figure 2:**
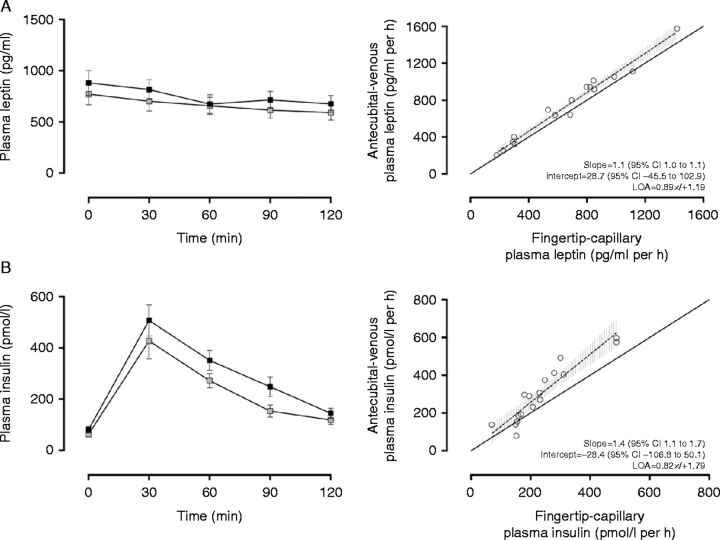
(A) Comparison of plasma leptin (pg m/l) measures obtained simultaneously from fingertip-capillary and antecubital-venous blood samples (left). Deming regression scatter plot (right). The solid line represents the line of equality. The dashed line denotes the regression line with corresponding 95% CIs represented in the grey hashed area. (B) Comparison of plasma insulin (pmol/l) obtained from simultaneous fingertip-capillary and antecubital-venous blood samples (left). Deming regression scatter plot (right). The solid line represents the line of equality. The dashed line denotes the regression line with corresponding 95% CIs represented in the grey hashed area. All values are expressed as mean±s.e.m. Grey shaded boxes represent values obtained from fingertip-capillary blood sampling, whereas shaded boxes represent concentrations in antecubital-venous blood.

Deming regression analysis revealed no evidence of systematic (intercept (95% CI)=−5.8 (−31.0 to 19.5)) or proportional bias (slope (95% CI)=1.1 (0.8–1.3)) between antecubital-venous (mean±s.d., 90.1±23.8 pg/ml per h) and fingertip-capillary (90.0±37.2 pg/ml per h) time-averaged AUC estimates of plasma glucagon (Fig. 1A). Similarly, Deming regression analysis of fingertip-capillary and antecubital-venous measures of plasma GLP1_7–36_ (mean±s.d., 8.6±3.4 pg/ml per h vs 9.1±3.0 pg/ml per h respectively, Fig. 1B) and leptin (mean±s.d., 664.5±350.3 pg/ml per h vs 741.0±375.2 pg/ml per h respectively, Fig. 2A) demonstrated no evidence of systematic or proportional bias between methodological approaches. For insulin, Deming regression revealed no evidence of systematic bias (intercept (95% CI)=−28.4 (−106.8 to 50.1)) between antecubital-venous (mean±s.d., 302.4±154.7 pmol/l per h) and fingertip-capillary (mean±s.d., 236.2±113.0 pmol/l per h) blood sampling; however, there was a proportional difference between measurements at higher concentrations (slope (95% CI)=1.4 (1.1 to 1.7, intercept (95% CI)=−28.4 (−106.8 to 50.1), Fig. 2B).

Agreement between fingertip-capillary and antecubital-venous-derived measures of glucagon (CV_a_=21.0%, LOA ±31.5 pg/ml per h, Fig. 1A) was good and modest for GLP1_7–36_ (CV_a_=24.0%, LOA ±2.5 pg/ml per h, Fig. 1B) and leptin (CV_a_=9.0%, LOA ×/÷ 1.19, Fig. 2A). For insulin, although the pattern of response to the standardised meal was similar, agreement between fingertip-capillary and antecubital-venous estimates was poor (CV_a_=36.0%, LOA×/÷1.79, Fig. 2B).

### Between-day reproducibility of fingertip-capillary-derived peptide hormones

**Figure 3:**
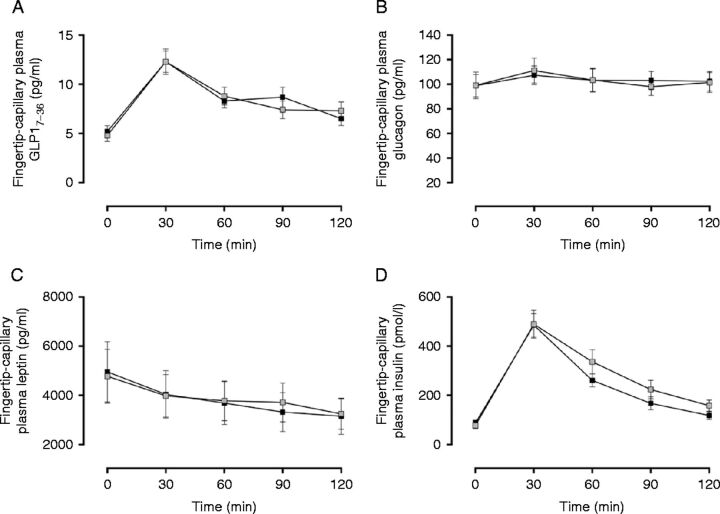
(A) Comparison of fingertip-capillary-derived measures of plasma GLP1_7–36_ (pg m/l) between days. (B) Comparison of plasma glucagon (pg m/l) between days. (C) Comparison of plasma leptin (pg m/l) between days. (D) Comparison of plasma insulin (pmol/l) between days. All values are expressed as mean±s.e.m. Grey shaded boxes represent values obtained from fingertip-capillary blood during visit 1, whereas shaded boxes represent concentrations in fingertip-capillary blood during visit 2. To convert GLP1_7–36_ (pg m/l), plasma glucagon (pg m/l) to their corresponding SI units multiply values by 0.298 and 0.287 respectively.

No systematic bias existed for time-averaged AUC measurement of any fingertip-capillary-derived peptides between visits (*P*≥0.05 for all). Reproducibility of plasma glucagon was strong between visits (mean±s.d., 103.3±36.9 pg/ml per h vs 103.6±32.9 pg/ml per h, CV_r_=8.2%, Fig. 3B). Plasma GLP1_7–36_ (8.6±3.2 pg/ml per h vs 8.8±3.0 pg/ml per h, Fig. 3A) and leptin (3870.8±3482.7 pg/ml per h vs 3774.9±3813.3 pg/ml per h, Fig. 3C) demonstrated modest reproducibility (CV_r_=22.7 and 25.0% respectively). Plasma insulin exhibited the greatest variability (291.7±142.1 pmol/l per h vs 253.9±94.7 pmol/l per h, CV_r_=36.0%, Fig. 3D), indicating a large degree of random error between visits.

## Discussion

This study is the first to examine the agreement between fingertip-capillary and antecubital-venous blood sampling for the determination of plasma GLP1_7–36_, glucagon, insulin and leptin, and the between-day reproducibility of these hormonal peptides measured utilising fingertip-capillary blood. The results presented here support the use of fingertip-capillary-derived estimates of glucagon, and to a lesser extent for GLP1_7–36_ and leptin. Caution should be exercised when utilising fingertip-capillary blood sampling for insulin quantification. The presence of proportional bias between fingertip-capillary and antecubital-venous estimates of insulin suggests these methods should not be employed interchangeably. These results have important implications for researchers and practitioners who wish to quantify the expression of these appetite- and metabolism-related peptides in various populations or field-based scenarios where antecubital-venous blood sampling might be contraindicated. Furthermore, fingertip-capillary blood sampling offers many advantages including simplistic application, cost/time effectiveness and reduced volume of blood required for analysis (6).

In this study, plasma glucagon and GLP1_7–36_ illustrated no evidence of systematic or proportional bias between methodological approaches, and no systematic bias was detected for between-day fingertip-capillary sampling, suggesting fasting and postprandial concentrations of these peptides are comparable between methodological approaches (Fig. 1) and reproducible between visits (Fig. 3). These observations coupled with the overall bias generated between fingertip-capillary and antecubital-venous time-averaged AUC for glucagon (−0.2 pg/ml per h) and GLP1_7–36_ (0.4 pg/ml per h) and between-day fingertip-capillary sampling (0.3 pg/ml per h and 0.2 pg/ml per h respectively) were not deemed clinically relevant discrepancies as based on our pre-determined values (4.9 pg/ml per h (22) and 1.4 pg/ml per h (2) respectively). Between methodological approaches, the typical percentage error (CV_a_) (%) of plasma glucagon and GLP1_7–36_ was similar (CV_a_=21.0 and 24.0% respectively), demonstrating modest agreement. Under test–rest conditions, fingertip-capillary-derived estimates of glucagon displayed strong reproducibility with an acceptable level of random error (CV_r_=8.2%), yet remained similar for plasma GLP1_7–36_ (CV_r__=_22.7%). The results concerning plasma glucagon are considerably lower than the biological variation presented by Widjaja *et al*. (25). Widjaja *et al*. (25) assessed the variation (within- and between-subject variation over 12 consecutive days) of several biochemical variables in healthy adults, and illustrated that antecubital-venous plasma glucagon exerted a daily variation of 19.0 and 28.0% within- and between-subjects respectively (25). Results of the current study may therefore suggest that estimates of fingertip-capillary plasma glucagon are comparable with their antecubital-venous equivalents, and more reliable when obtained within fingertip-capillary blood. To the authors' knowledge, this is the first study to report the test–retest reproducibility of plasma GLP1_7–36_. GLP1 is produced and secreted into the circulation from the L cells of the intestinal mucosa in response to meal ingestion, and is recognised as a key effector in glucose regulation, gastrointestinal motility and appetite (26, 27). In this study, plasma GLP1_7–36_ demonstrated moderate CV values with negligible bias between methodological approaches and days. Similar to plasma glucagon, results of the current study may suggest that estimates of GLP1_7–36_ are more reliable when obtained within fingertip-capillary blood. Nonetheless, further research quantifying the biological variation of GLP1_7–36_ is warranted to elucidate the within- and between-subject variation of this peptide. Taken together, fingertip-capillary blood sampling provides reliable and comparable measures of glucagon and GLP1_7–36_ with antecubital-venous samples, indicating the two methodological approaches may be interchanged. These results are of importance as they permit comparison of results obtained in studies utilising fingertip-capillary blood sampling with studies using antecubital venous blood sampling.

Although Deming regression revealed no evidence of systematic or proportional bias between methodological approaches (Fig. 2A), and no systematic bias for between-day fingertip-capillary sampling, estimates of fingertip-capillary plasma leptin were consistently underestimated in comparison with their antecubital-venous equivalents. It might be unsurprising that the concentrations of leptin differed between methodological approaches given its production site, manner of collection and differing sampling location. Leptin is predominantly secreted from the adipose tissue (28), whereas all other hormones analysed within this study are secreted from either the pancreas or the gastrointestinal tract. The antecubital vein drains a mixture of forearm muscle, adipose and skin tissues (29), compared with the fingertip, which drains an assortment of blood from venules, arterioles, intracellular and interstitial fluid, and of course the capillaries. The concentrations of plasma leptin may therefore be more pronounced in venous outflow, as illustrated in this study. Despite this tendency, the biases generated between methodological approaches (−77.5 pg/ml per h, CV_a_=9.0%) and between days (95.9 pg/ml per h) were not deemed clinically relevant discrepancies and likely to alter research interpretation based on our pre-determined values (148.0 pg/ml per h), which takes into account the within-subject daily variation of plasma leptin (24). Under test–reset conditions, fingertip-capillary-derived estimates of leptin demonstrated modest reproducibility, with a satisfactory typical percentage error (CV_r_=25.0%). Leptin, a tonic adipocyte hormone, indicates long-term energy balance (30, 31) and signals chronic nutritional state. It may be plausible that the increased bias and level of typical error between days may be attributable to dietary standardisation. In this study, dietary standardisation was only implemented from the evening meal preceding data collection periods. Standardisation to promote identical nutritional states may therefore require longer dietary replication. Nonetheless, the typical error reported between days was similar to that reported in earlier investigations (24, 32). These researchers established the reproducibility of fasting plasma leptin among lean and obese individuals in antecubital-venous blood, and provided evidence that plasma leptin exerts a daily variation of 20% (24). Based on the typical percentage error, we report that fingertip-capillary blood sampling is an acceptable approach for leptin quantification, but should not be interchanged with antecubital-venous blood sampling.

For insulin quantification, Deming regression revealed evidence of proportional difference between fingertip-capillary and antecubital-venous blood (slope=1.4, 95% CI for slope=1.1 to 1.7, 95% CI for intercept=−106.8 to 50.1), and visual inspection of scatter plots indicated worse agreement between measurements at higher concentrations (Fig. 2B). The identification of proportional bias between fingertip-capillary and antecubital-venous blood sampling illustrates that these techniques cannot be used interchangeably. The typical percentage error of plasma insulin was similar between methodological approaches and between visits (CV_a_ and CV_r_=36.0% respectively), representing a large level of random error. The CV values demonstrated here are higher than the within-subject variation of plasma insulin (26%) as illustrated by Widjaja *et al*. (25). Despite this, the bias generated between days (−37.8 pmol/l per h) was not deemed a clinically relevant discrepancy according to our pre-determined values (62.0 pmol/l per h), which took into account the within-subject daily variation of plasma insulin. Nonetheless, compared with antecubital-venous blood, Deming regression, typical percentage error and LOA suggest that fingertip-capillary represents an inappropriate alternative for insulin quantification. Perhaps fingertip-capillary blood, being arterialised, is comparable and more reflective of arterial-derived estimates. Further work to elucidate this is necessary. Researchers and practitioners wishing to implement this technique should take the random error between techniques into consideration when interpreting study findings.

No data concerning differences in any of the peptides reported here based on antecubital-venous and fingertip-capillary blood sampling exist. The error observed (differences observed for each analyte between methodological approaches and between visits) may be attributable to pre-analytical (e.g. specimen collection, timing of collection) and analytical error (e.g. collection techniques, sample handling). As mentioned, blood attained following fingertip-capillary blood sampling is characteristically dissimilar to that of antecubital-venous blood, and may be more reflective of arterial blood (12). Furthermore, discrepancies between fingertip-capillary and antecubital-venous-derived peptides might have been influenced by the introduction of cytoplasmic matter (intracellular fluid) to the fingertip-capillary sample. Excessive squeezing during fingertip-capillary blood sampling encourages haemolysis of the erythrocytes, thus influencing the introduction of intracellular contents to the surrounding blood plasma consequently diluting samples and affecting assay effectiveness (33). In this study, highly trained research staff collected all fingertip-capillary blood samples and care was taken to avoid excessive pressure whilst sampling in an attempt to minimise the risk of such error.

This study provides valuable information for researchers and practitioners regarding the utility of fingertip-capillary blood sampling for the estimation of appetite- and metabolism-regulating peptides. Caution should be observed when extrapolating the results of this study, as the findings might be limited to relatively short durations (2 h) and a healthy adult population. Further research is required to quantify the agreement for longer duration sedentary periods, in other situational states and alternative populations. It is of great importance to note that the results presented here can only be assumed for samples taken as EDTA plasma with the respective additives (aprotinin (33 μl/ml blood) and a di-peptidyl peptidase IV inhibitor (30 μl/ml blood)). Furthermore, the authors’ acknowledge that values for CV% in the current study appear relatively high, particularly for GLP1_7–36_ and leptin, yet are consistent with the variability expected between plasma samples from the same sampling location. For this reason, the findings of this study suggest that fingertip-capillary blood sampling represents a suitable and comparable alternative to antecubital-venous methods for the estimation of certain appetite-related peptides.

In conclusion, this study demonstrates that at rest fingertip-capillary blood sampling offers an appropriate methodological and reproducible approach for the quantification of glucagon and to a lesser extent for GLP1_7–36_ and leptin. Caution should be exercised when using fingertip-capillary sampling for plasma insulin quantification in repeated measures designs, and should not be interchanged with antecubital-venous blood sampling. These findings will allow for appropriate comparison of capillary and venous sampling techniques in the fields of appetite and metabolism, where fingertip-capillary sampling might offer a more appropriate approach (e.g. paediatrics, adolescents and elderly).

## Declaration of interest

The authors declare that there is no conflict of interest that could be perceived as prejudicing the impartiality of the research reported.

## Funding

The Dairy Council UK externally funded the present study.

## Author contribution statement

B P Green, P L S Rumbold, and E Stevenson helped to design the study. B P Green and J T Gonzalez performed all data collection and biochemical analysis. K Thomas and B P Green conducted the statistical analysis for the study. J T Gonzalez, K Thomas, P L S Rumbold, and E Stevenson critically reviewed drafts of the manuscript and provided assistance with its preparation. B P Green wrote the final manuscript.
